# Rehabilitation towards functional independence in patient with abdominal tuberculosis undergone abdominal surgery: a case report

**DOI:** 10.11604/pamj.2022.41.195.33026

**Published:** 2022-03-10

**Authors:** Shivani Satish Lalwani, Moli Jai Jain, Vishnu Diwakar Vardhan, Vaishnavi Dilip Yadav, Tasneem Mustafa Lakkadsha, Sakina Shoeb Hussain Saifee

**Affiliations:** 1 Ravi Nair Physiotherapy College, Datta Meghe Institute of Medical Sciences, Sawangi (M), Wardha, Maharashtra, India

**Keywords:** Abdominal surgery, small bowel obstruction, abdominal tuberculosis, physiotherapy management, case report

## Abstract

A small bowel obstruction is one of the most prevalent life-threatening situations. The most common clinical signs are vomiting, stomach discomfort, abdominal distension, and severe constipation. A 23-year-old girl presented to the multispecialty hospital with stomach pains that had persisted for two days. The patient experienced identical issues two months earlier and was treated conservatively. Radiography in the manner of abdominal X-ray and ultrasound were used to appropriately diagnose intestinal blockage. She underwent exploratory laparotomy for the same. Numerical pain rating scale, Incentive Spirometer (IS), mobility scale, anxiety and depression scale, independence measure were used as an outcome measure. Medical management was successful, but to return the patient to her normal daily routine activities without signs of dyspnea or early fatigue following abdominal surgery, a comprehensive rehabilitation program incorporating various respiratory techniques was developed, which proved to be effective and correlated with medically substantial gains in physical functioning and wellbeing.

## Introduction

Intestinal blockage occurs when the forward movement of digestive materials is obstructed at any point along the gastrointestinal system's length, such as by a little or big bowel obstruction [1]. The most common clinical characteristics are bilious vomiting, stomach discomfort, abdominal distension, bloating, and extreme constipation. The etiology of been varied with adhesions in 60%, strangulated hernia in 20%, malignancy in 5%, and volvulus in 5% [2]. In India, tuberculosis of the abdomen causes around 3% to 20% of all intestinal blockages [3]. These patients underwent exploratory laparotomy and may be accompanied by various postoperative pulmonary complications (PPCs). PPCs are due to disturbance of normal respiratory muscle action following anesthesia induction, which also increases hospital morbidity, lengthens hospital stays, and adds to the expense of health care. These complications further contribute to respiratory dysfunction, causing a long-term decline in functional residual and vital capacity [4,5].

We present a case of a 23-year-old girl who has undergone abdominal surgery requiring efficient physiotherapy rehabilitation to speed up recovery by preventing or resolving PPCs and providing physical rehabilitation to help patients restore to their premorbid level. Key measurements involve respiratory function, early mobility, and pain reduction, with quality of life also being assessed.

## Patient and observation

**Patient information:** on the 20^th^ of November 2021, a 23-year-old girl presented to the multispecialty hospital with complaints of abdominal pain for the past two days that were subtle at the beginning and eventually evolved in nature, as well as vomiting (6-7 episodes) and constipation for the past two days. Soft widespread soreness was found across the abdomen during inspection and palpation.

The patient gave a history of identical concerns experienced two months ago, on September 10^th^, 2021, where she was treated conservatively. There was ultrasonography performed, and the results indicated a thickened edematous transverse and descending colon with paraaortic lymphadenitis, referring to Koch's. On the same day, the patient was diagnosed with abdominal tuberculosis and has been on anti-tuberculosis therapy for the past two months. The patient gave a history of amenorrhea for the past two months, as well as a loss of weight and appetite. Following examination, she has advised admission in the Surgery Intensive Care Unit (SICU) for surgery after being diagnosed with small intestinal obstruction following a series of tests. She had explorative laparotomy under general anesthesia on November 21, 2021, with a midline incision over the abdomen, resection, and anastomosis of the small bowel.

**Clinical findings:** on postoperative day 1, with the patient's agreement, for the clinical examination, she was positioned in a half-lying position with sufficient back support. On inspection, Foley´s catheter, Ryle´s tube, and the abdominal drain were in situ at the left iliac region. The movement of the chest wall was noted to be reduced throughout the inspection along with engagement of the accessory muscles of inspiration. Cardiovascular findings were determined to be normal on examination, with a pulse rate of 90 beats/minute and blood pressure of 108/77 mmHg, respectively. Breathing was a normal and thoracoabdominal type, with a rate of 17 breaths per minute. On palpation, the examination findings were confirmed: diminished chest excursion was observed on both the right and left sides due to soreness over the incision site on the abdomen. At the axillary, nipple, and xiphisternum phases, chest expansion revealed differences of 2 cm, 2 cm, and 1 cm, respectively. A 16-centimeter-long midline incision ran from the xiphoid process to the umbilicus. In both lung fields, auscultation demonstrated reduced air entry. The patient was having respiratory muscle weakness, early exhaustion on the activity of daily living.

**Timeline of the current episode:** this is illustrated in Table 1.

**Table 1:** entire sequence of events

Date	Events
10th September 2021	First time USG (ultrasonography) was done
20th November 2021	The diagnosis was done
20th November 2021	Admission date
20th November 2021	Second time USG was done
21st November 2021	Laparotomy surgery was done
21st November 2021	ICD was inserted
22nd November 2021	Physiotherapy examination was done
27th November 2021	ICD was removed
6th December 2021	Discharge date

USG: ultrasonography; ICD: implantable cardioverter defibrillator

**Figure 1:**
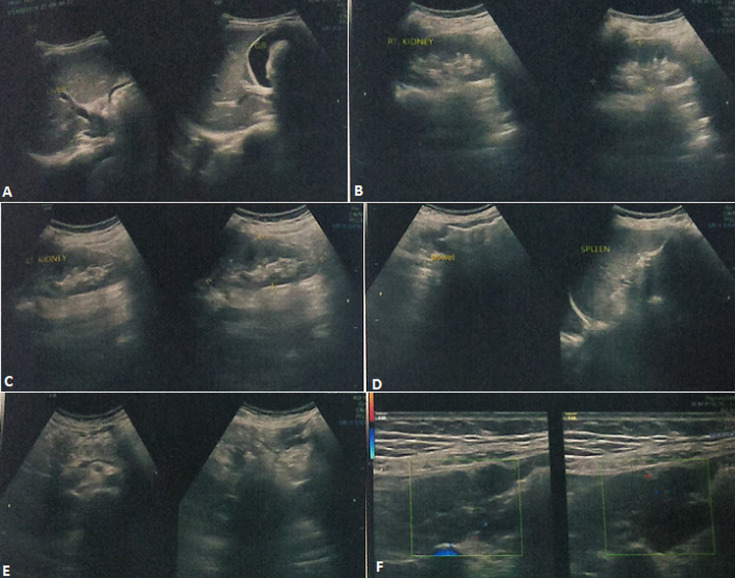
(A, B, C, D, E, F) ultrasound image taken for the first time on September 10, 2021

**Diagnostic assessment:** pre-operative ultrasonography (USG), first-time was done on 10^th^ September 2021 of abdomen reveals thickened edematous transverse and descending colon with paraaortic lymphadenitis reference of Kochs (Figure 1). The patient underwent repeat USG was on 20^th^ November 2021 of the abdomen which suggestive of free fluid in the abdomen with multiple reverberation artifacts throughout the abdomen and the presence of a perforation. Visualized bowel on linear probe shows the thickened wall.

**Figure 2:**
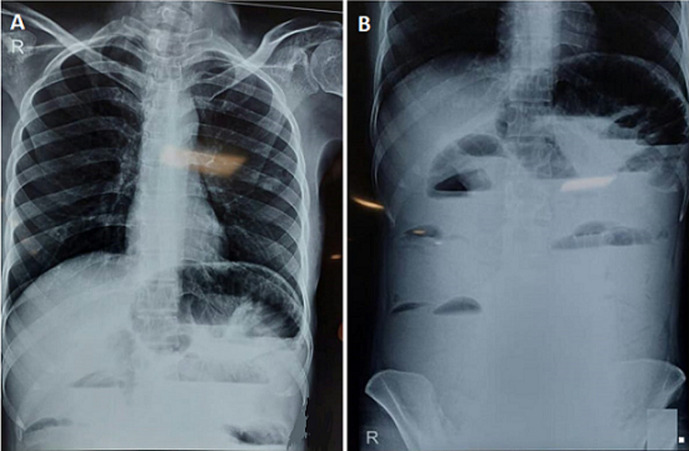
A) chest X-ray showing radiolucent gastric bubble over the left side; B) X-ray of abdomen showing multiple small bowel loops and one large bowel loop suggestive of obstruction distal to the transverse colon

Along with this chest X-ray (Figure 2A) was done which shows a radiolucent gastric bubble over the left side and an X-ray of the abdomen (Figure 2B) showed multiple small bowel loops and one large bowel loop suggestive of obstruction distal to the transverse colon. Post operatively, lab investigations were done and the following derangements were observed, Complete Blood Count (CBC) - reduced hemoglobin (7.3 gm/dl Hb), Kidney Function Test (KFT) - urea level raised (47) and sodium level decreased (134), Liver Function Test (LFT) - albumin level decreased (1.8), Random Blood Sugar (RBS) -glucose-plasma random - 66mg percent (decreased).

**Diagnosis:** small bowel obstruction underwent explorative laparotomy.

**Therapeutic interventions:** the objective of this patient's physiotherapy treatment was to let her return to her everyday activities with the least amount of weariness and shortness of breath possible. Our objective was to ease dyspnea, reduce discomfort, enhance breathing, induce relaxation, and improve overall functional status while keeping the patient's goals in mind. Physiotherapeutic therapies were given to this patient for two weeks.

**Figure 3:**
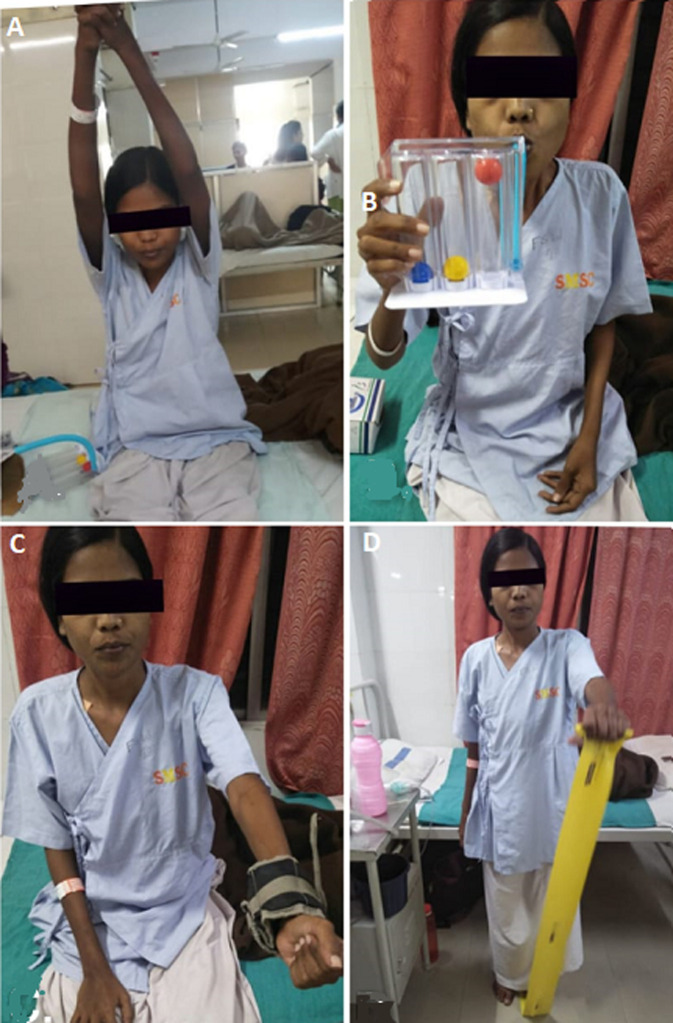
interventions provided in week 1: A) patient is performing thoracic expansion exercise; B) patient is performing Incentive spirometry; C) patient is performing strength training with a weight cuff; D) patient is performing strength training with Thera band

**Figure 4:**
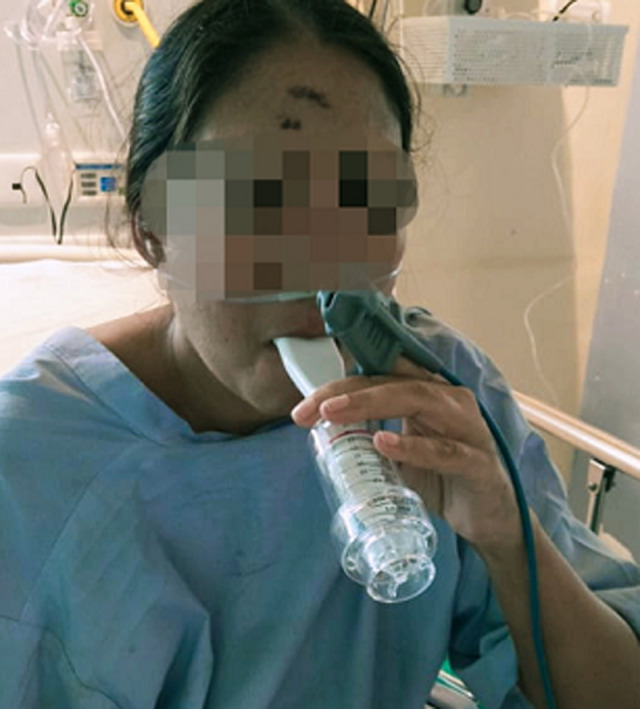
inspiratory muscle training to the patient given in week 2

**Physiotherapy treatment goals:** these are shown in Table 2, Table 3, Figure 3 and Figure 4.

**Table 2:** interventions provided in week 1

Sr. No.	Physiotherapy treatment goals	Therapeutic intervention	Treatment regimen
**1**.	To provide awareness of the condition, gain co-operation and consent of the patient and his family members	Patient and caregiver education and counselling about the exercise regimen and the importance of adherence to it	Patients and caregivers were educated about the importance of positioning every 2 hourly, early ambulation, and activity of daily living
**2**.	To prevent pulmonary, circulatory and integumentary complications post-surgery	Manual Positioning: half lying/semi-fowlers position was given initially; later upright sitting was given; air beds provided; ankle pumps	Positioning was given after every 2 hours: initially 10 reps x 1 set 2 times a day; later 10 reps x 2 sets 3-4 times a day
**3**.	To reduce pain at the incision site	pain control modality - TENS 4 electrodes were placed -2 above the umbilicus, 2 below para-medially	TENS therapy was administered for one minute at a stimulation level of 40-60 milliamperes
**4**.	To avoid strain over incision and suture site	Abdominal binders	Binder support during movements
**5**.	To improve bed mobility and to prevent prolonged immobilization	Monitored in bed transitional training and bedside mobilization given with binder: from rolling to side-lying, sitting, supported standing, standing, and spot marching was given	1-3 days: rolling, side-lying; 4-8 days: sitting and supported standing; 9th day onwards: standing and spot marching
**6**.	To promote airway clearance	1) Manual chest percussion and vibrations; 2) manually assisted cough	1) For 1-3 days initially; 2) for 4-8 days
**7**.	To improve breathing patterns, reduce dyspnea and respiratory rate	Deep breathing exercises: 1) Diaphragmatic breathing; 2) 4-4-8 breathing; 3) segmental breathing	Initially 10 reps x 1 set 2 times a day; later 10 reps x 2 sets 3-4 times a day
**8**.	To improve lung volumes (IRV) and capacities (FRC)	1) Thoracic expansion exercises: shoulder in full flexion with deep inspiration and extension with expiration	Initially 10 reps x 1 set 2 times a day; later 10 reps x 2 sets 3-4 times a day.
		2) Incentive spirometry (initially): flow-oriented spirometer used. Visual Feedback through different balls representing 600,900 and 1200cc	Starting from third-day post-surgery; initially 2-3 times a day; later in every 2 hours of interval
**9**.	To maintain joint integrity and mobility and prevent joint stiffness	AROM exercises of upper and lower limbs bilaterally	Initially 10 reps x 1 set 2 times a day; later 10 reps x 2 sets 3-4 times a day
**10**.	To bring back to normal ADL's	Self-paced walking in 30 meters hallway	Begin on post-op day 4, initially, 5 min, progressing up to 15-20 min

TENS: transcutaneous electrical nerve stimulation; IRV: inspiratory reserve volume; FRC: functional residual capacity; AROM: active range of motion; ADL's: activities of daily living

**Table 3:** interventions provided in week 2

Treatment from week 1 was continued, along with additional interventions, in week 2			
Sr No.	Physiotherapy treatment goals	Therapeutic intervention	Treatment regimen
**1**.	To promote airway clearance	Active cycle of breathing technique (ACBT)	Eigth day onwards
**2**.	To maintain muscle power and endurance and to prevent muscle wasting	Upper and lower limb strengthening: bridging; knee rolling; Thera band strengthening; weight cuff training	Started after 1-week post-surgery 10 reps x 1 set 2 times a day
**3**.	To improve respiratory muscle strength (mechanical breathing device)	Threshold inspiratory muscle trainer (IMT)	After 1-week post-surgery IMT training begins

**Figure 5:**
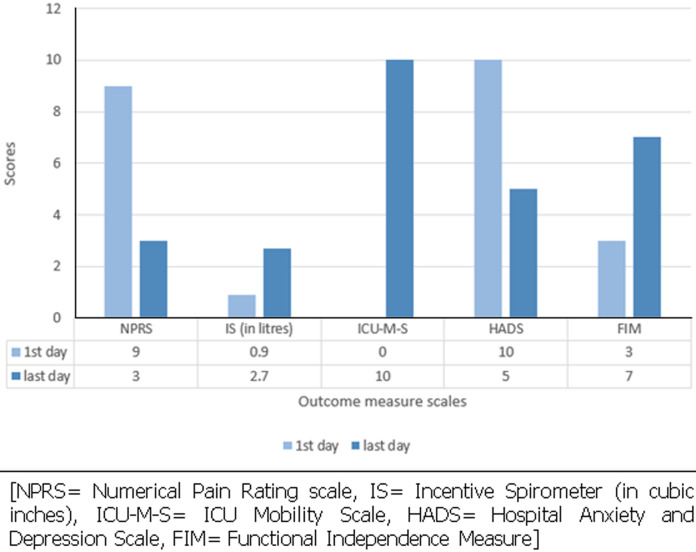
pre and post physiotherapy rehabilitation, scores on outcome measure response

**Follow-up and outcome of interventions:** this is illustrated in Figure 5.

**Patient perspective:** during the therapy session, the patient was optimistic and receptive. She was delighted with her progress and was prepared to continue as long as the sessions were satisfactory.

**Informed consent:** written informed consent was collected from the patient's parents and the patient herself. The patient was pleased and satisfied with her progress.

## Discussion

Following abdominal surgery, chest physiotherapy has generally been a routine part of post-operative treatment, intending to prevent or minimize problems such as atelectasis, pneumonia, sputum retention, and restrictive lung pattern with alterations in pulmonary mechanics [6]. Other postoperative physiotherapy management includes early mobilization, splinted coughing or huffing, active cycle of breathing technique (ACBT), and the use of different mechanical devices. From the present study, we concluded that flow-oriented Incentive spirometry proved to be beneficial in improving pulmonary function (FVC) as well as functional capacity [7].

Post-discharge therapeutic interventions may improve long-term results since abdominal surgery impacts physical recovery and health-related quality of life. Nonetheless, post-major abdominal surgery rehabilitation is still in its early phases [8]. Another perk of physiotherapy is that it can help with continence, particularly during coughing and powerful expiratory movements. As there are negative consequences of extended bed rest, all patients after any sort of abdominal surgery should begin ambulation as soon as safely practicable [9]. Physical activity once or twice a day for up to 15-30 minutes is both safe and effective for critically sick patients, according to a growing body of evidence [8]. Inspiratory muscle training (IMT) is an efficient preparatory physiotherapy technique for reducing postoperative pulmonary complications (PPC) as well as the length of hospitalization (LOS) following serious surgery. The IMT dosage is connected to the efficiency of physiotherapy therapies; prescriptions should aim for at least two weeks of supervised sessions lasting more than 15 minutes, with enforced load increments and sufficient abdominal support over, as well as the incorporation of additional exercise modalities [10].

In this case study, our goal is to speed up patients´ recovery by avoiding or resolving postoperative pulmonary complications (PPCs) and providing physical rehabilitation to help patients return to premorbid status. A two-week physiotherapy regimen was given to the patient, which had a positive influence on the patient's condition.

## Conclusion

The pulmonary rehabilitation program has been demonstrated to be beneficial and has been linked to statistically significant increases in exercise tolerance and wellbeing. This case study offers an integrated plan for the rehabilitation of patients post abdominal surgery. Full recovery of the patient was not achieved during the rehabilitation program, but most of the therapeutic objectives were achieved during this plan, such as improved breathing pattern, increased functional vital capacity, reducing pain, improving chest expansion and ADLs of the patient after 2 weeks of intensive physiotherapeutic intervention.
